# Gut microbiome as a response marker for pancreatic enzyme replacement therapy in a porcine model of exocrine pancreas insufficiency

**DOI:** 10.1186/s12934-020-01482-2

**Published:** 2020-12-03

**Authors:** Sabrina Ritz, Daniela Hahn, Haleluya T. Wami, Karin Tegelkamp, Ulrich Dobrindt, Juergen Schnekenburger

**Affiliations:** 1 Biomedical Technology Center of the Medical Faculty, University of Muenster, Mendelstrasse 17, 48149 Muenster, Germany; 2 Institute of Hygiene, University of Muenster, 48149 Muenster, Germany

**Keywords:** Exocrine pancreatic insufficiency, Gut microbiota, α-diversity, β-diversity, Pancreatic enzyme replacement therapy

## Abstract

**Background:**

Exocrine pancreatic insufficiency (EPI) is characterized by the loss of active pancreatic enzymes and a resulting severely reduced food digestion. EPI therapy requires orally applied pancreatic enzyme replacement. The gut microbiome is a known mediator of intestinal diseases and may influence the outcome of EPI and the effects of a pancreatic enzyme replacement therapy (PERT). Here, we analyzed the effects of EPI and PERT on the gut microbiome in the model of pancreatic duct ligated minipigs.

**Results:**

The microbial community composition in pig feces was analyzed by next generation sequencing of 16S rRNA amplicons. The data were evaluated for α- and β-diversity changes and changes at the different Operational Taxonomic Unit (OTU) levels by Shannon–Wiener and inverse Simpson index calculation as well as by Principal Coordinates Analysis based on Bray–Curtis dissimilarity. Microbial α-diversity was reduced after EPI induction and reverted to nearly healthy state after PERT. Analysis of microbial composition and β-diversity showed distinctive clusters of the three study groups and a change towards a composition comparable to healthy animals upon PERT. The relative abundance of possible pathobionts like *Escherichia*/*Shigella*, *Acinetobacter* or *Stenotrophomonas* was reduced by PERT.

**Conclusion:**

These data demonstrate that EPI-induced dysbiosis could be reverted by PERT to a nearly healthy state. Elevated α-diversity and the reduction of bacterial overgrowth after PERT promises benefits for EPI patients. Non-invasive microbiome studies may be useful for EPI therapy monitoring and as marker for response to PERT.

## Background

Exocrine pancreatic insufficiency (EPI) is a severe impairment of food digestion as a result of a loss of active pancreatic enzymes. EPI is defined as a decline of pancreatic enzyme release to less than 5–10%. It can be induced by several disorders like cystic fibrosis (CF), acute (AP) or chronic pancreatitis (CP), pancreatic cancer, diabetes mellitus or as a complication of gastrointestinal surgery [1–5]. EPI patients are affected by weight loss and malnutrition [4, 6]. EPI symptoms significantly impair the quality of life, and malnutrition especially of fat soluble vitamins subsequently may result in other severe diseases like osteoporosis, osteopenia or low-trauma fracture [7, 8]. Disease progression, severity and occurring comorbidities in the course of EPI strongly varies between patients and correlates with the etiology of pre-existing diseases and lifestyle of the affected person (e.g. nutrition, smoking, alcohol consumption etc.) [6].

EPI treatment is mainly based on the orally applied pancreatic enzyme replacement therapy (PERT) [6, 9]. Due to the heterogeneous course of the disease and the wide range of symptom manifestations, the oral dose of enzymes has to be adjusted to every patient individually. Other effects observed in CF as well as in CP, are small intestinal bacterial overgrowth (SIBO) and changes in the microbial gut composition that in turn leads to worsening of the symptoms [10, 11]. One possible cause for this is a food oversupply in the intestine by the lack of digestion in the host and the lack of pancreatic juice with its antimicrobial function [12]. SIBO and the associated reduction of microbial diversity (i.e. α-diversity) as well as loss of beneficial bacteria are hallmarks of existing dysbiosis [13].

In humans the bacterial diversity increases after birth and has its first climax approx. at the age of 3 years [14]. The total number of bacteria of an adult is estimated to be 3.8*10^13^ [15] and there is a permanent interaction between microorganisms and the host [13]. Progress in both next-generation sequencing as well as big data handling enables increasing insights into the bacterial colonization of the gastrointestinal tract. As a result, statements about non-culturable bacteria and thus the composition and distribution within the bacterial community could be made [16]. The microbiome gets more and more into the focus of investigations to shed light on the role of the intestinal bacteria on the severity of EPI and a possible impact on therapy.

A model to study the course of EPI and the influence of EPI on cleavage and absorption of food is the ligation of the pancreatic duct in minipigs [2]. Because of the high similarity in digestion compared to humans and the feasibility to ligate the solitary and separated pancreatic duct, pigs and especially minipigs are preferred as EPI models. Using minipigs, the impact of pancreas duct ligation on digestion and absorption of metabolites could be shown [2, 17]. Furthermore, the effect of PERT was investigated in EPI pigs and an improvement of this type of therapy could be achieved [18].

Our aim was to study the influence of PERT on the gut microbiome in pancreatic duct ligated minipigs. Therefore, we performed a longitudinal analysis of the composition of the intestinal bacterial community via 16S rRNA amplicon sequencing to analyze the direct effect of an induced EPI and supplemented pancreatic enzymes in EPI. By monitoring the change of the intestinal microbiome caused by the functional loss of the exocrine pancreas and the influence of PERT, new possible therapy targets, such as the manipulation of the microbiome by antibiotics or microbiome transfer, easily accessible endpoints for disease state and therapy monitoring and possibly markers for the efficacy of PERT as a microbiome pattern might be found in the future.

## Results

The effects of an experimental EPI and PERT on the intestinal microbiome composition was analyzed in the Göttingen minipig model by next generation sequencing of 16S rRNA amplicons from pig feces. The data were evaluated for α- and β-diversity changes and changes at the different OTU levels. Samples from healthy animals were provided by the breeder and reflect the microbiome status at the delivery of the animals.

### Alpha diversity

**Fig. 1:**
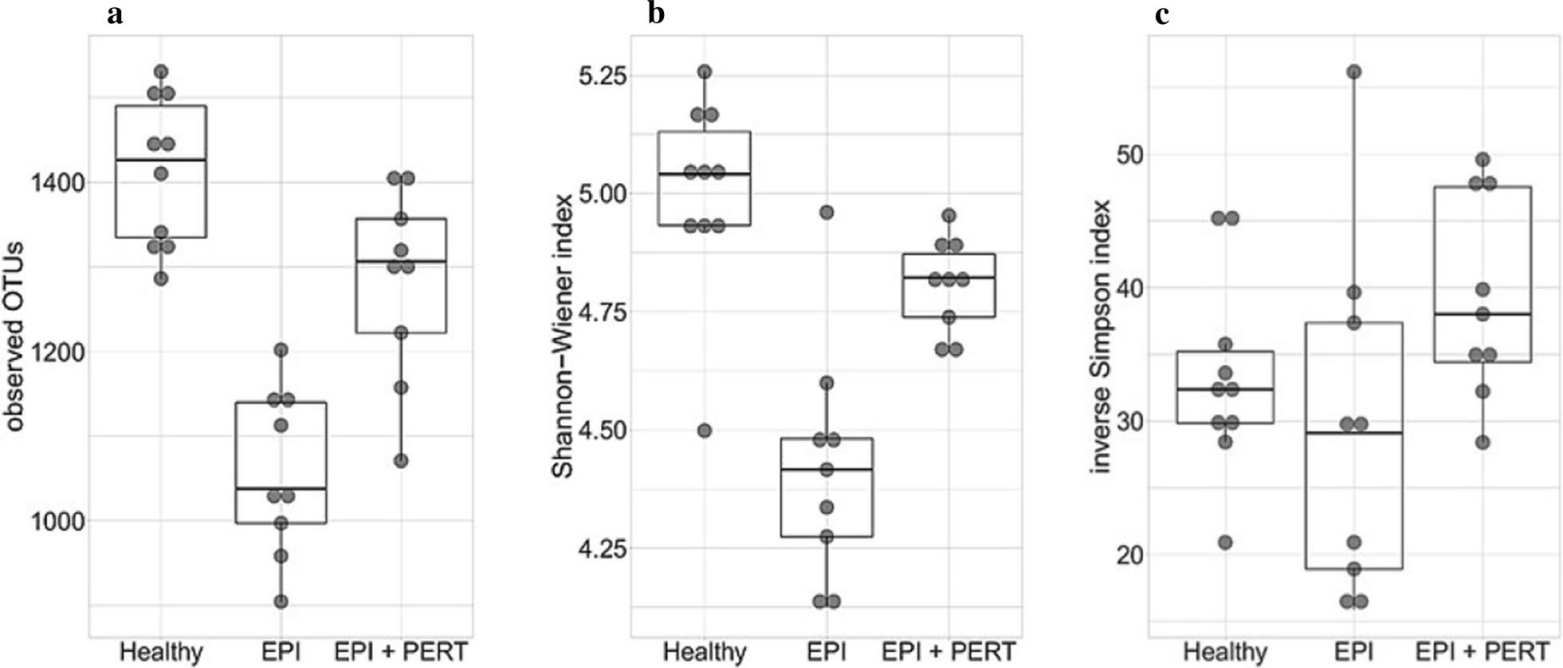
α-diversity of gut microbiota in healthy Göttingen minipigs (Healthy, n = 10), Göttingen minipigs with induced exocrine pancreatic insufficiency (EPI, n = 9) without and with pancreatic enzyme replacement therapy (EPI + PERT, n = 9). Richness and evenness were analyzed via the number of observed Operational Taxonomic Units (OTUs) (**a**), Shannon–Wiener index (**b**) and inverse Simpson index (**c**)

To investigate the impact of the substitution of pancreatic enzymes in a porcine EPI model on gut microbial composition, we analyzed the species richness of the gut microbiota in Göttingen minipigs. The α-diversity Shannon–Wiener index as well as the inverse Simpson index were estimated from the number of observed Operational Taxonomic Units (OTUs). The number of observed OTUs reflects richness, whereas the Shannon–Wiener, as well as inverse Simpson index, evaluate both, richness and evenness. Although the last mentioned indices are both heterogeneity measures, the Shannon–Wiener index places more emphasis on rare species and the inverse Simpson index places more emphasis on dominant species [19]. The calculated values of these indices correlate with diversity. Thus, higher values indicate both more existing species (i.e. richness) and a more even distribution (i.e. evenness) of these species in the microbial composition. The α-diversity regarding the number of observed OTUs (Fig. 1a) and Shannon–Wiener index (Fig. 1b) were highest in the group of healthy animals (1412 ± 84 and 5.0 ± 0.20, respectively) and lowest in animals with EPI (1058 ± 93 and 4.42 ± 0.24, respectively). The α-diversity differs significantly between the healthy animals and the EPI affected animals regarding observed OTUs (*p* < 0.001) and Shannon–Wiener index (*p* < 0.001). The treatment of the EPI pigs with enzyme substitution led to a significant increase of α-diversity regarding the number of observed OTUs (1282 ± 106, *p* = 0.00015) and Shannon–Wiener index (4.81 ± 0.09, p = 0.003), and a tendency towards α-diversity levels of the healthy animals was observed. The dominance-oriented inverse Simpson index (Fig. 1c) showed the highest value for the treated animals with EPI (39.29 ± 7.15), followed by the healthy animals (33.36 ± 7.02), and animals with EPI without treatment (29.51 ± 12.49). However, no significant difference was found.

### Microbial composition

**Fig. 2:**
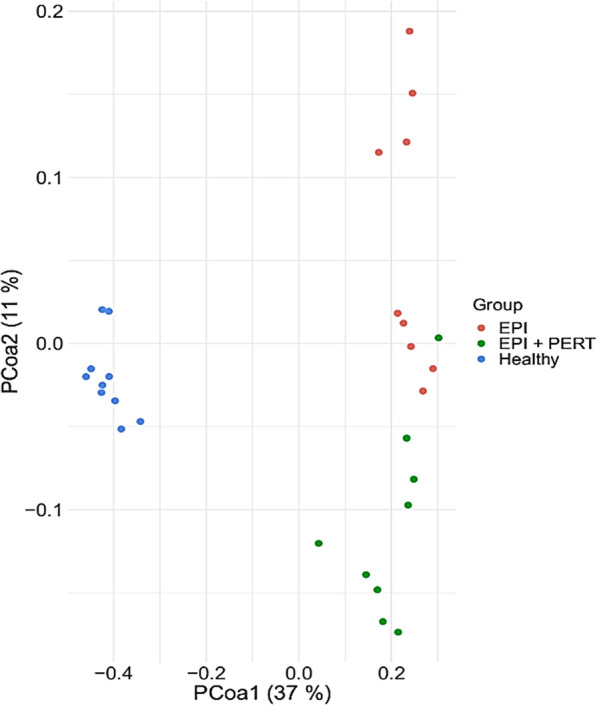
β-diversity between gut microbiota of healthy Göttingen minipigs (Healthy, n = 10), pancreas duct-ligated Göttingen minipigs without treatment (EPI, n = 9) or with pancreatic enzyme replacement therapy (EPI + PERT, n = 9) visualized as a PCoA plot. Values are calculated via the Bray–Curtis dissimilarity coefficient

We also analyzed the differences amongst the gut microbial composition in more detail. The bacterial composition differed significantly between healthy animals, animals affected by EPI and affected animals treated with PERT (AMOVA, *p* < 0.05). Regarding β-diversity visualized in the Principal Coordinates Analysis (PCoA) plot based on the Bray–Curtis dissimilarity coefficient, healthy pigs showed narrower clustering than animals affected by EPI (Fig. 2). It should be noted that the distance between the data points of this figure correlates with the similarity of the microbial composition of the corresponding animals. This means that samples that are close to each other in this figure also show a higher similarity to each other than samples whose data points are further apart. Although pigs suffering from EPI showed a broader distribution, a tendency towards a defined clustering could be seen and the centers of both clusters differed significantly (AMOVA, *p* = 0.014) indicating that treating the EPI pigs with PERT led to a uniform change in the microbial composition.

**Fig. 3:**
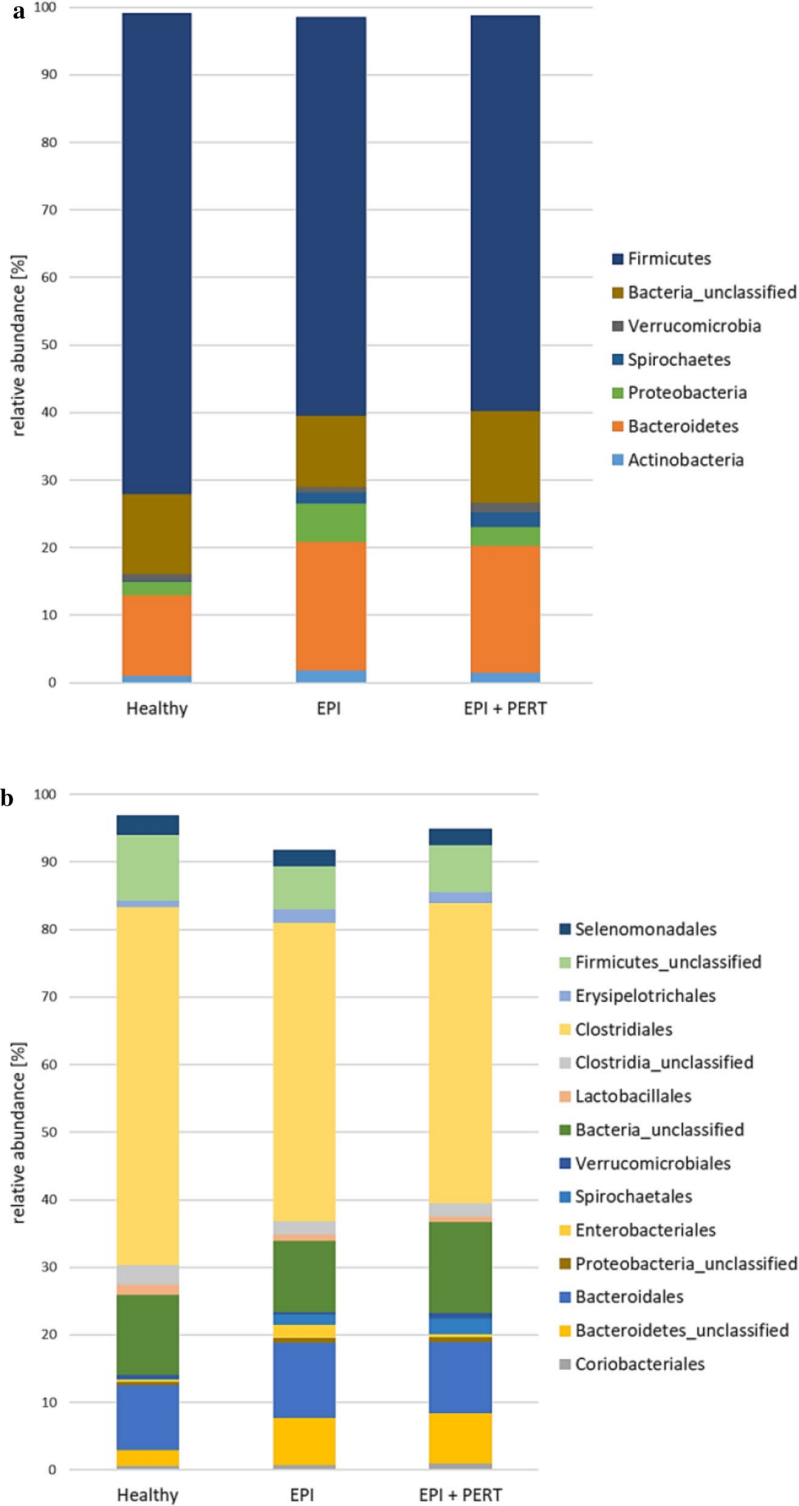
Relative abundance of bacterial phyla (**a**) and bacterial orders (**b**) in healthy Göttingen minipigs (Healthy, n = 10), in Göttingen minipigs with induced exocrine pancreatic insufficiency without treatment (EPI, n = 9) or after 28 days pancreatic enzyme replacement therapy (EPI + PERT, n = 9). Mean values > 0.5% of relative abundance are shown

The differences in β-diversity were also seen at the level of relative abundance of the bacterial genera. The relative abundance of bacterial phyla and order with a value > 0.5% are shown in Fig. 3, where a decrease in the phylum *Proteobacteria* (Fig. 3a) and the order *Enterobacteriales* and an increase in the order *Spirochaetales* (Fig. 3b) could be seen after PERT (for further details on bacterial order and family see Additional file 1: A, B).

**Fig. 4:**
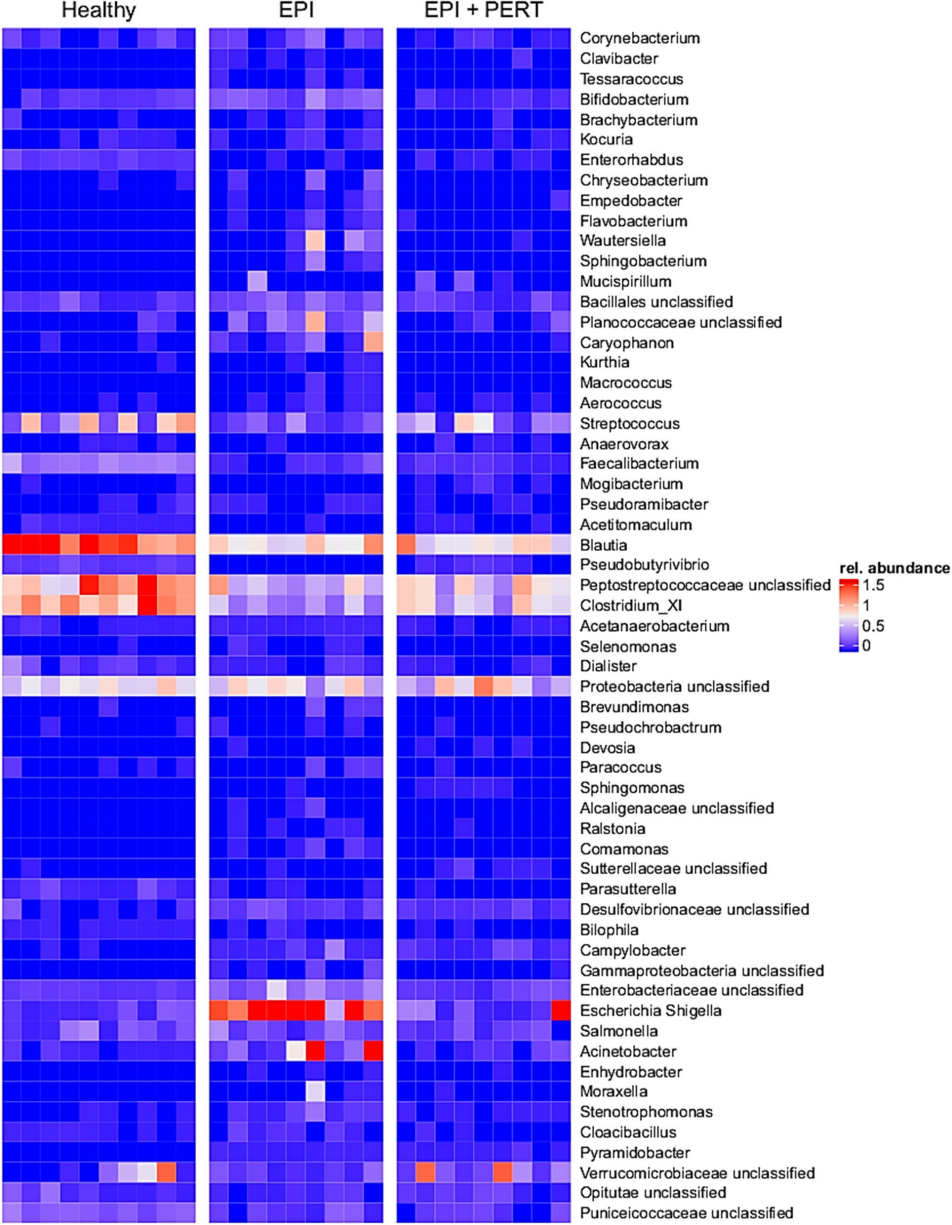
Relative abundance of bacterial genera in healthy Göttingen minipigs (Healthy, n = 10), in Göttingen minipigs with induced exocrine pancreatic insufficiency without treatment (EPI, n = 9) or after 28 days pancreatic enzyme replacement therapy (EPI + PERT, n = 9). OTUs with altered relative abundance after treatment are shown. Red color represents highest relative abundance, blue color represents lowest relative abundance

In Fig. 4, the relative abundance of OTUs with changed prevalence is depicted. OTUs with ≥ 0.05% abundance are shown. PERT considerably altered the bacterial composition (Tables 1 and 2) and a tendency towards the abundance pattern of the healthy animals can be assumed (Fig. 4, Additional file 2). Most genera had a remarkably decreased relative abundance (Table 1) and only the presence of 5 genera was increased (Table 2). Besides these alterations in relative abundance, a change in the pattern of microbial community after PERT and a tendency of development towards the detected pattern of the bacterial composition of the healthy animals could be seen (Additional file 2). Measured relative abundance of all detected OTUs are shown in Additional file 2 (for further details see Additional file 2: A–D).

**Table 1:** Decreased abundance in percent of bacterial genera in pancreatic duct-ligated Göttingen minipigs compared to PERT treated animals

Decreased bacterial genera	EPI		EPI + PERT		Δ	p	p_cor_
	Med.	IQR	Med.	IQR			
*Corynebacterium*	0.127	0.137	0.041	0.066	− 65.25	0.024	0.179
*Brachybacterium*	0	0.049	0	0	− 64.41	0.009	1.725
*Kocuria*	0.053	0.086	0	0.043	− 65.75	0.009	0.862
*Tessaracoccus*	0	0.058	0	0	− 100	0.009	0.575
*Bifidobacterium*	0.191	0.089	0.051	0.051	− 68.98	0.009	0.431
*Prevotella*	2.420	0.855	1.895	1.036	− 26.95	0.024	0.172
*Sphingobacterium*	0	0.077	0	0	− 100	0.009	0.345
*Chryseobacterium*	0	0.147	0	0	− 100	0.009	0.288
*Empedobacter*	0	0.050	0	0	− 69.84	0.009	0.246
*Wautersiella*	0	0.287	0	0	− 96.75	0.043	0.285
*Brevundimonas*	0	0.104	0	0	− 100	0.009	0.216
*Ralstonia*	0	0.049	0	0	− 78.54	0.009	0.192
*Comamonas*	0.040	0.078	0	0	− 100	0.009	0.172
*Parasutterella*	0	0.047	0	0	− 80.91	0.043	0.275
*Bilophila*	0	0.042	0	0.019	− 53.39	0.009	0.157
*Escherichia/Shigella*	1.375	0.634	0.096	0.296	− 80.35	0.013	0.099
*Acinetobacter*	0.264	1.632	0.076	0.123	− 92.89	0.009	0.144
*Enhydrobacter*	0	0.047	0	0	− 71.62	0.009	0.133
*Stenotrophomonas*	0.090	0.067	0.043	0.027	− 61.57	0.043	0.266
*Cloacibacillus*	0.053	0.101	0	0.021	− 77.08	0.009	0.123
*Caryophanon*	0.053	0.182	0	0	− 97.26	0.009	0.115
*Kurthia*	0	0.045	0	0	− 100	0.009	0.108
*Macrococcus*	0	0.052	0	0	− 100	0.009	0.101
*Pseudoramibacter*	0.047	0.055	0	0.039	− 59.74	0.043	0.257
*Acetanaerobacterium*	0.047	0.010	0.043	0.048	− 37.61	0.009	0.096
*Dialister*	0.048	0.063	0.038	0.053	− 40.09	0.009	0.091
*Selenomonas*	0	0.055	0	0	− 100	0.009	0.086

Bacterial genera with a noteworthy reduced abundance after 28 days of PERT based on the Wilcoxon signed-rank test (α = 0.05) are listed

EPI: relative abundance in EPI affected pigs; EPI + PERT: relative abundance in EPI pigs after treatment; med.: median; IQR: interquartile range; Δ: relative change based on original mean abundance; p:  p-value; p_cor_: p-values corrected according Benjamini–Hochberg method

All values are in percent and genera are shown in taxonomic order

**Table 2:** Increased abundance in percent of bacterial genera in pancreatic duct-ligated Göttingen minipigs compared to PERT treated animals

Increased bacterial genera	EPI		EPI + PERT		Δ	p	p_cor_
	Med.	IQR	Med.	IQR			
*Sphingomonas*	0	0	0	0.042	443.03	0.009	0.082
*Streptococcus*	0.106	0.145	0.335	0.536	149.42	0.043	0.249
*Mogibacterium*	0	0	0.038	0.045		0.009	0.078
*Pseudobutyrivibrio*	0	0	0.049	0.084		0.009	0.075
*Clostridium_XI*	0.271	0.254	0.639	0.374	73.89	0.033	0.223

Bacterial genera with a noteworthy increased abundance after 28 days of PERT according to the Wilcoxon signed-rank test (α = 0.05) are listed

EPI: relative abundance in EPI affected pigs; EPI + PERT: relative abundance in EPI pigs after treatment; med.: median; IQR: interquartile range; Δ: relative change based on original mean abundance; p: *p*-value; p_cor_: *p*-values corrected according Benjamini–Hochberg method

All values are in percent and genera are shown in taxonomic order

**Fig. 5:**
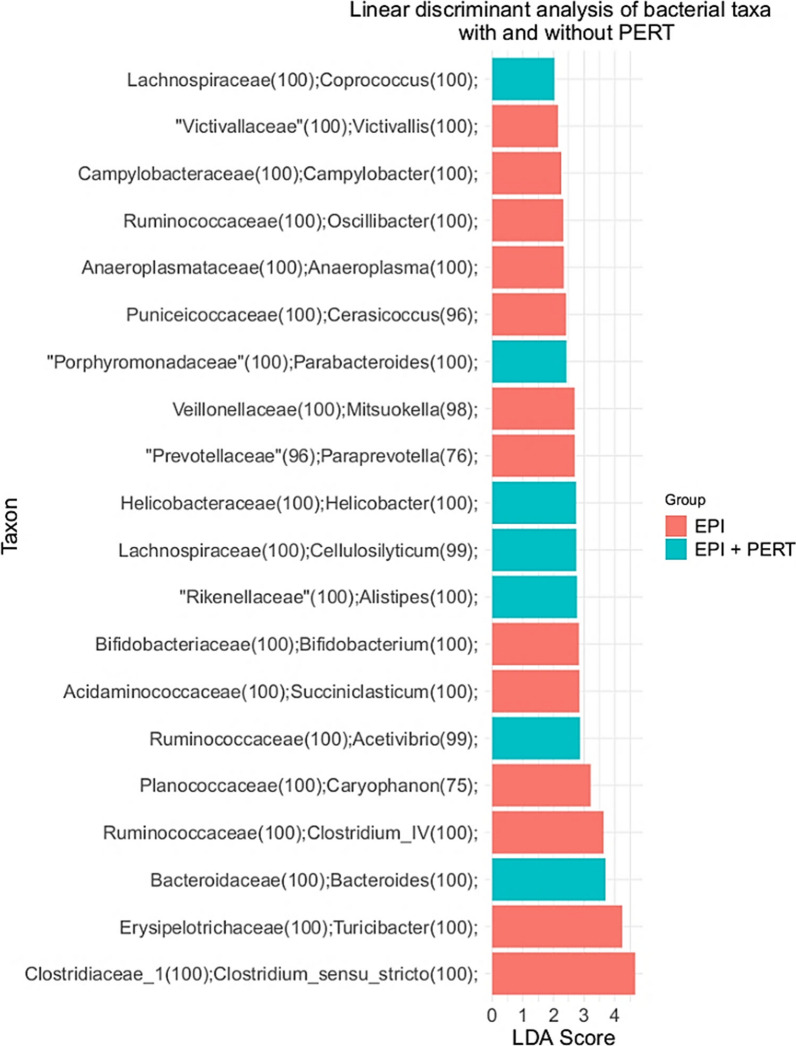
Linear discriminant analysis effect size (LEfSe) based on unique sequences found in pancreatic duct-ligated Göttingen minipigs without treatment (EPI, n = 9) or after pancreatic enzyme replacement therapy (EPI + PERT, n = 9). LDA score shows the impact of the OTU on separability between EPI and EPI + PERT. (LDA score = 2.0, α = 0.05)

The following comparison of microbial composition refers to EPI-affected animals, as the healthy animals had different husbandry conditions and therefore a direct comparison between the healthy and EPI affected pigs would not be appropriate. Linear discriminant analysis effect size (LEfSe) was used to determine the OTUs that most likely accounted for the differences (i.e. separability of both groups) between the bacterial composition of the treated and the untreated EPI animals (Fig. 5). In this algorithm, OTUs are determined with unique sequences, which only occurred in one group of animals [20]. The calculated linear discriminant analysis (LDA) score correlates with the impact of the respective OTU on the differential bacterial composition of EPI pigs before and after treatment.

The LDA score reflects an impact on separability by the genera *Coprococcus*, *Parabacteroides*, *Helicobacter*, *Callulosilyticum*, *Alistipes*, *Acetivibrio* and *Bacteroides* in the group of PERT-treated animals with EPI. On the other hand, the genera *Victivallis*, *Campylobacter*, *Oscillibacter*, *Anaeroplasma*, *Cerasiococcus*, *Mitsuokella*, *Paraprevotella*, *Bifidobacterium*, *Succiniclasticum*, *Caryophanon*, *Clostridium IV*, *Turicibacter* and *Clostridium* *sensu stricto* are discriminants of the bacterial community composition of the untreated EPI animals. One has to take into account that although these unique sequences of the respective OTUs concisely distinguish the bacterial colonisation of the animals before and after treatment, no predication about the relative abundance of the respective genera can be made.

## Discussion

The direct impact of exocrine pancreas insufficiency on the gut microbiota and the subsequent effects of pancreatic enzyme replacement therapy has been poorly studied so far. Because of various factors that affect bacterial composition and variety of EPI–causing diseases, a direct link between the functional loss of the exocrine pancreas activity and of microbiota within the gastrointestinal tract is missing. Here, we established an EPI model in 10 female Göttingen minipigs and analyzed the effect of EPI and PERT on the gut microbiota. So far, our study is the first to describe the intestinal microbiota after PERT in a standardized model and thus the microbiota were unaffected by previous diseases and the associated therapies. The structure of the study was designed in a way, that other influencing factors (e.g. sex, mode of delivery, age, feed, and housing) could be neglected. As expected, the induction of EPI by pancreatic duct ligation reduced α-diversity and affected the composition of the intestinal microbiome. In the EPI minipigs, we observed two major findings: the α-diversity within each animal was elevated after treatment with PERT while the bacterial composition changed remarkable after treatment. We could detect a decreased abundance of *Corynebacterium, Brachybacterium, Kocuria, Tessaracoccus, Bifidobacterium, Prevotella, Sphingobacterium, Chryseobacterium, Empedobacter, Wautersiella, Brevundimonas, Ralstonia, Comamonas, Parasutterella, Bilophila, Escherichia/Shigella, Acinetobacter, Enhydrobacter, Stenotrophomonas, Cloacibacillus, Caryophanon, Kurthia, Macrococcus, Pseudoramibacter, Acetanaerobacterium, Dialister and Selenomonas* as well as increased abundance of *Sphingomonas, Streptococcus, Mogibacterium, Pseudobutyrivibrio and Clostridium_XI*. In general, a reduced bacterial diversity (i.e. α-diversity), as well as a changed microbial composition, are hallmarks of dysbiosis.

A correlation between dysbiosis and various diseases such as obesity, autism, Crohn´s disease as well as ulcerative colitis has already been observed several times [21–24]. Our findings indicate that PERT could restore the intestinal microbiome composition to a nearly healthy state resembling the bacterial colonisation of healthy animals. After PERT, the EPI-affected pigs had a significantly increased α-diversity (p < 0.01) measured by observed OTUs and Shannon–Wiener index. Regarding the inverse Simpson index the treated EPI pigs displayed even a higher bacterial diversity than the healthy pigs. The differences in assessing richness and evenness within the animals (Shannon–Wiener index and inverse Simpson index) could be due to the focus on rare (Shannon–Wiener index) or dominant species (inverse Simpson index) of the respective algorithm used. The Shannon–Wiener index of the EPI pigs was significantly increased after treatment, so that possibly an augmented frequency of rare bacteria instead of dominant bacteria could be assumed. Because of several changes in digestion (i.e. lack of pancreatic enzymes, higher acidity, diminished motility etc.) caused by EPI, there is an oversupply of food for dominant bacterial genera and a lack of host defence. In a recent study in humans, it was shown that mainly the altered function of pancreatic acinar cells, rather than the absence of pancreatic juice, causes an alteration in gut microbiome and a reduction in α–diversity [25]. Therefore, an overgrowth of dominant bacteria can be expected. The importance of antimicrobial peptides secreted by acinar cells has already been demonstrated in a mouse model [26]. In this study, animals showed bacterial overgrowth after deletion of ion channel Orail in pancreatic acinar cells, leading to systemic infection and death within 2 weeks. The survival rate of these animals was significantly improved by the administration of the cathelicidin-related antimicrobial peptide (CRAMP) [26]. Furthermore, the changed intestinal environment caused by EPI could promote the rise of pathobionts. An increased occurrence of previously reduced bacteria resulting from the decline of dominant bacteria after PERT and PERT -associated reduction in food oversupply could therefore be regarded as a beneficial effect, and it is conceivable that the reported worsening of the symptoms, as it occurs in EPI-associated diseases like CP or CF [10, 11], could be prevented or alleviated by this medication. A directional change in the bacterial composition was also apparent in the representation of the β-diversity calculated by the Bray–Curtis dissimilarity coefficient. Here, the EPI-affected pigs showed a distinct clustering and the centers of the clusters differ significantly with p = 0.014 (AMOVA).

Only a few reports studying the effects of EPI and PERT on the gastrointestinal microbiome composition of animal models are available. An alteration of the composition of intestinal microbiota after supplementation of pancreatic digestive enzymes was observed in healthy mice [27]. In this study, an advantageous effect of PERT was assumed to rely on the increased relative abundance of beneficial bacteria e.g. *Akkermansia muciniphila* or *Lactobacillus reuteri*. This is in good agreement with the increase of *A. muciniphila* after PERT detected in our study in EPI pigs (Additional file 2: C). Remarkably, besides the increased abundance of some beneficial bacteria, most genera showed a diminished relative abundance. This effect supports the hypothesis that bacterial overgrowth was induced by the ligation of the pancreatic duct and the subsequent lack of the antimicrobial function of pancreatic juice [28] in our minipig study.

Our and the aforementioned study had controversial outcomes regarding the specific bacterial composition and this may be due to the different model organism, nutrition and the fact, that the PERT-treated mice were healthy. Healthy adult animals exhibit most probably a different homeostasis of the gut microbiota. An interesting finding is the failure of enzyme supplementation to affect the α-diversity in healthy mice. This indicates that PERT may only be beneficial in the case of a dysbiosis.

Similar results of altered microbial compositions were seen in a study in which healthy dogs and PERT-treated and untreated EPI-affected dogs were compared [29]. Here, a non-significant trend regarding elevated α-diversity in the diseased cohort of dogs after PERT was observed. The elevated α-diversity in EPI pigs upon PERT seen in our study is in accordance to these findings. The comorbidity of bacterial overgrowth, as it can be assumed for the EPI pigs, was also seen in human cystic fibrosis patients and host-mediated inflammation [10, 30]. In the course of intestinal inflammation and the following dysbiosis, an overgrowth with pathobionts, especially members of the γ-proteobacteria, was observed and the importance of a balanced gut microbiome for the maintenance of a healthy gut was emphasized [30]. Furthermore, the change in the proportion of members of the phylum *Proteobacteria* in the intestinal microbiome has also been discussed as a potential diagnostic marker, because of its correlation to dysbiosis in the sense of pathologic imbalance of the intestinal microbiota and subsequent diseases [31]. Beside bacterial overgrowth, an increase in the genus *Prevotella*, which is known to be correlated with chronic inflammation, was observed in a mouse-study as well as in humans with reduced exocrine pancreatic function [25, 26].

A decreased relative abundance of pathogens of the genus *Prevotella* as well as of the phylum Proteobacteria and especially in the class of γ-Proteobacteria (i.e. genera *Escherichia/Shigella*, *Acinetobacter*, *Stenotrophomonas*) could be seen in our study under PERT, and the reduced prevalence of these potential pathobionts may suggest a beneficial effect of PERT in EPI-affected individuals (Fig. 3a, Table 1).

Currently, there has been no direct link between specific bacterial strains or a distinct bacterial composition and the etiology or severity of EPI. Though, a correlation between dysbiosis and the severity of acute pancreatitis (AP), one of many pre-existing diseases of EPI, was already described in patients as well as in mice with induced AP [32]. As mentioned before, one parameter describing dysbiosis is the α-diversity, which was significantly reduced in humans affected by AP. The reduced α-diversity of EPI pigs in our study is consistent with these findings. Furthermore, the increased relative abundance of *Escherichia/Shigella* in mice with induced AP correlated positively with the ability of the bacteria to invade epithelial cells and therefore with the aggravation of the disorder from mild to severe form of AP. The subsequent deteriorated barrier function results in bacterial translocation, which in turn causes the progression of the systemic inflammatory response [32]. In this context, the increased α-diversity as well as the reduced relative abundance of *Escherichia/Shigella* in our study implies that the frequency of infections caused by these invasive bacteria should also decrease.

As a result of these findings the reduced relative abundance of possible pathobionts like *Escherichia/Shigella*, *Acinetobacter* or *Stenotrophomonas* in combination with an increased α-diversity could function as a marker for the therapeutic success of PERT. Microbiome studies may be useful for EPI therapy monitoring and disease state evaluation with a non-invasive and low burden method.

In most of the EPI-causing pre-existing diseases (i.e. cystic fibrosis, acute and chronic pancreatitis), an impaired intestinal barrier, inflammation and/or imbalanced immune homeostasis are major factors for the progression and the severity of the diseases [33–35]. In the aforementioned studies, disease severity and progression were correlated with dysbiosis marked by a lower α-diversity [30, 32]. With regard to this, a higher α-diversity as seen after PERT in our study can be seen as a positive effect and, in the long run, a progression of the EPI-related symptoms and comorbidities could be possibly delayed or stopped.

## Conclusion

Our study is the first that describes the effect of supplementation with pancreatic enzymes in an induced exocrine pancreatic insufficiency model in minipigs. Because of the higher similarity to humans compared to other laboratory animals regarding digestion [2], our findings may help to elucidate the ongoing discovery of new potential treatment targets to improve the health status of EPI patients and to reduce present comorbidity. Monitoring of the microbiome particularly with regard to α-diversity during PERT could support therapy dose finding and thus show more quickly whether the correct enzyme dose has been found or whether further action is required. The observed elevated α-diversity and the reduction of bacterial overgrowth after PERT promises benefits for patients. In conclusion, the investigation of the intestinal microbiome could be a potent diagnostic tool and promises, after further experimental validation, to facilitate the monitoring of the disease and to possibly improve the therapeutic success of PERT.

## Materials and methods

### Animals and sample collection

Study design includes a cohort of ten female Göttingen Minipigs (age 3–4 years). The phenotype of exocrine pancreatic insufficiency (EPI) in the animals was induced by a surgical ligation of the pancreatic duct 30 months before PERT. The pigs were housed in groups of two within the Central Animal Facility (ZTE) of the University Hospital Münster. Altromin^®^ 9050/51 (Lage, Germany) was fed twice a day without (“EPI”) or with (“EPI + PERT”) 2 g of pancreatic enzymes added (Kreon, activity 50,000 μ/g, Abbott Laboratories GmbH, Hannover, Germany). At the beginning of the study as well as after 28 days with treatment, feces was collected from every animal for 3 days and directly stored at 4 °C after collection. For every animal, samples were pooled and stored at − 20 °C. As reference, feces of 10 adult female Göttingen Minipigs (“Healthy”) was used (kindly provided by the breeder Ellegaard Göttingen Minipigs: Ellegaard Göttingen Minipigs, Dalmose, Denmark).

### Isolation of DNA from feces and 16S rRNA sequencing

For DNA extraction, fecal samples were thawed at room temperature. DNA was extracted using the ZymoBIOMICS DNA Miniprep Kit (Zymo Research Europe GmbH, Freiburg, Germany) and stored at − 80 °C until further use. Since an insufficient amount of DNA could be isolated from a sample from one EPI pig, the samples from this animal were excluded from further analysis. 16S rRNA amplicon libraries were created according to the Illumina 16S Metagenomic Library Preparation guidelines (Illumina Inc., San Diego, CA, USA). The V4 region of the bacterial 16S rRNA gene was amplified using the primer pair 515f (5′-TCGTCGGCAGCGTCAGAT GTGTATAAGAGACAGGTGYCAGCMGCCGCGGTAA-3′) and 806Rb (5′-GTC TCGTGGGCTCGGAGATGTGTATAAGAGACAGGACTACNVGGGTWTCTAAT-3′) with Illumina sequencing adaptors. The amplicon reactions were cleaned using Agencourt AMPure^®^ XP beads (Beckman Coulter, Krefeld, Germany) and an indexing PCR was performed with the Nextera XT Index Primer Set A (Illumina, Inc., San Diego, CA). Libraries were normalized to 4 nM, pooled, and paired-end sequenced (2 × 250 bp) with 5% PhiX samples on a Illumina MiSeq platform (Illumina, Inc., San Diego, CA) using v2 sequencing chemistry. We used the ZymoBIOMICS microbial Community DNA Standard (Zymo Research Europe GmbH, Freiburg, Germany) as a reference for validation of our microbiomic workflow.

### Data analysis

Obtained sequences were quality trimmed and analyzed by the mothur software package (v1.41.1) [36]. Following the Miseq SOP [37], the paired-end sequences were assembled into contigs. Afterwards, the sequences were screened and reads with ambiguous bases or more than 8 homopolymers were removed. The demultiplexed sequences were aligned to Silva database [38] (Release v.123) and chimeras were removed using the VSEARCH algorithm. Assignment of taxonomic classifications was done using the Wang approach and sequences classified as chloroplast, mitochondria, archaea and eukaryota were removed.

Subsequently, the classified bacterial sequences were assigned to Operational Taxonomic Units (OTUs) with a cutoff of 97% similarity using the opticlust algorithm. For further analysis of α- and β-diversity, a subsample was set to 47,405 sequences. α-diversity (i.e. richness and evenness) was calculated OUT-based via numbers of observed OTUs, Shannon–Wiener index, inverse Simpson index, and phylogeny based on rarefaction. β-diversity was analyzed using the Bray–Curtis dissimilarity coefficient. To analyze the effect of the conducted treatment of the animals, we used linear discriminant analysis effect size (LEfSe) within mothur to determine OTUs that most likely account for the separability of the treated and non-treated EPI animals. LDA score was set to 2.0 and the statistical parameters to α = 0.05, respectively.

### Statistical analysis

To analyze whether the α-diversity (i.e. observed OTUs, Shannon–Wiener index, and inverse Simpson index, respectively) differs between the microbial communities of the EPI animals with and without PERT, paired *t*-test was used with significance defined as *p* < 0.05. The differences between the healthy and the EPI affected animals was analysed by unpaired* t*-test. The differences regarding the bacterial composition at the genus level between these two animal groups was investigated using Wilcoxon signed-rank test (α = 0.05) and p-values were corrected with the Benjamini–Hochberg method to control the false discovery rate [39]. We calculated the mentioned statistical test with Microsoft Excel (Excel 2016 MSO) and considered only the animals with induced EPI. Since the healthy pigs were housed in another facility and fed with other fodder than Altromin^®^ 9050/51, a direct comparison between these animals and the animals with EPI was not appropriate and the statistical analysis was carried out only on the samples of animals with induced EPI before and after treatment.

To determine whether the centers of the cluster computed for every animal group differ significantly, we used analysis of molecular variance (AMOVA) within the mothur package.

## Supplementary information

**Additional file 1.**  Relative abundance of bacterial order (A) and family (B) in healthy Göttingen minipigs (Healthy, n = 10), in Göttingen minipigs with induced exocrine pancreatic insufficiency without treatment (EPI, n = 9) or after 28 days pancreatic enzyme replacement therapy (EPI + PERT, n = 9). Mean values > 0.5% of relative abundance are shown.

**Additional file 2.** Heat map of the relative abundance of microbial genera in healthy Göttingen minipigs (Healthy, n = 10), in Göttingen minipigs with induced exocrine pancreatic insufficiency without treatment (EPI, n = 9) or after at least 28 days pancreatic enzyme replacement therapy (EPI + PERT, n = 9). Red color represents highest relative abundance, blue color represents lowest relative abundance.

## Data Availability

The datasets used and/or analysed during the current study are available from the corresponding author on reasonable request.

## Abbreviations

AMOVA: Analysis of molecular variance
AP: Acute pancreatitis
CF: Cystic fibrosis
CP: Chronic pancreatitis
CRAMP: Cathelicidin-related antimicrobial peptide
EPI: Exocrine pancreatic insufficiency
LDA: Linear discriminant analysis
LEfSe: Linear discriminant analysis effect size
OTU: Operational Taxonomic Unit
PCoA: Principal Coordinates Analysis
PERT: Pancreatic enzyme replacement therapy
SIBO: Small intestinal bacterial overgrowth
ZTE: Central Animal Facility of the University Hospital Münster
