# Supplementary material for: Gut microbiome as a response marker for pancreatic enzyme replacement therapy in a porcine model of exocrine pancreas insufficiency
Source: Microb Cell Fact. 2020 Dec 3;19:221. doi: 10.1186/s12934-020-01482-2 (PMC7713139; doi:10.1186/s12934-020-01482-2)
Supplement: Supplementary file 1 — Additional file 1. Relative abundance of bacterial order (A) and family (B) in healthy Göttingen minipigs (Healthy, n = 10), in Göttingen minipigs with induced exocrine pancreatic insufficiency without treatment (EPI, n = 9) or after 28 days pancreatic enzyme replacement therapy (EPI + PERT, n = 9). Mean values > 0.5% of relative abundance are shown. [file 12934_2020_1482_MOESM1_ESM.docx]

**Additional file 1**

A

**
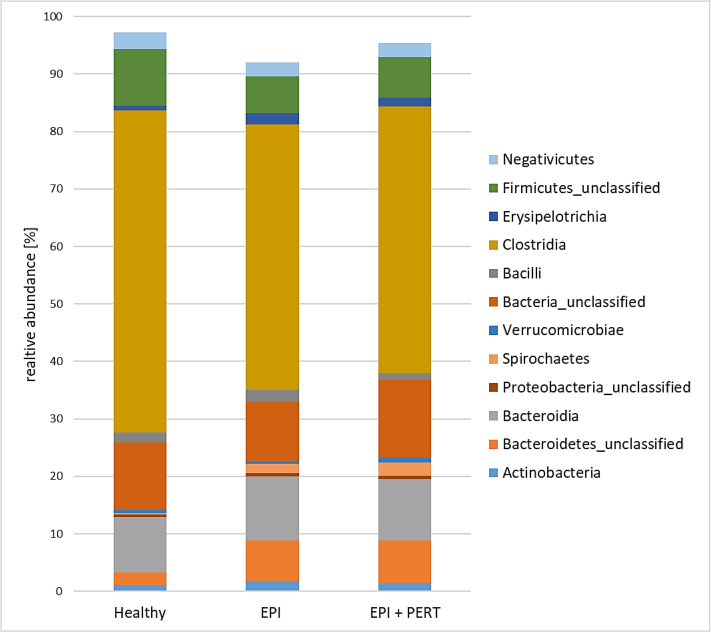
**

**
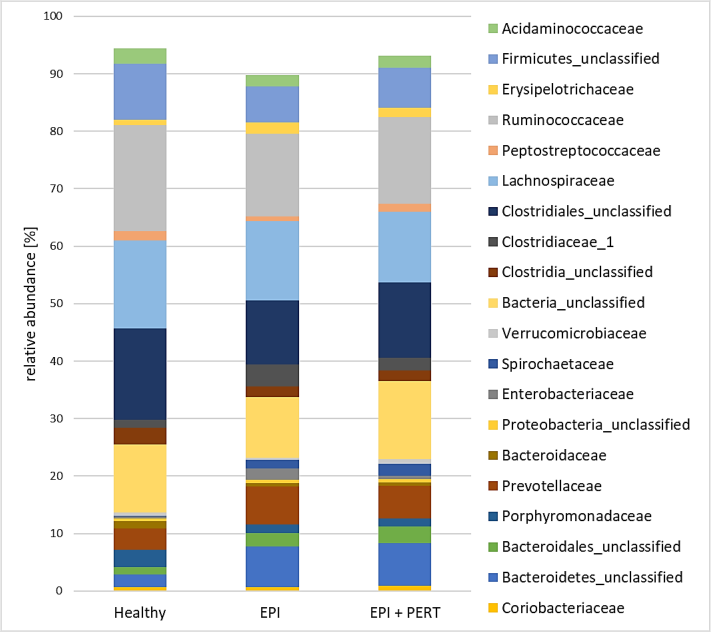
**

B

Realtive abundance of bacterial order (A) and family (B) in healthy Göttingen minipigs (Healthy, n = 10), in Göttingen minipigs with induced exocrine pancreatic insufficiency without treatment (EPI, n = 9) or after 28 day pancreatic enzyme replacement therapy (EPI + PERT, n = 9). Mean values > 0.5 % of relative abundance are shown.
