# Supplementary material for: Gut microbiome as a response marker for pancreatic enzyme replacement therapy in a porcine model of exocrine pancreas insufficiency
Source: Microb Cell Fact. 2020 Dec 3;19:221. doi: 10.1186/s12934-020-01482-2 (PMC7713139; doi:10.1186/s12934-020-01482-2)
Supplement: Supplementary file 2 — Additional file 2. Heat map of the relative abundance of microbial genera in healthy Göttingen minipigs (Healthy, n = 10), in Göttingen minipigs with induced exocrine pancreatic insufficiency without treatment (EPI, n = 9) or after at least 28 days pancreatic enzyme replacement therapy (EPI + PERT, n = 9). Red color represents highest relative abundance, blue color represents lowest relative abundance. [file 12934_2020_1482_MOESM2_ESM.docx]

**Additional file 2**


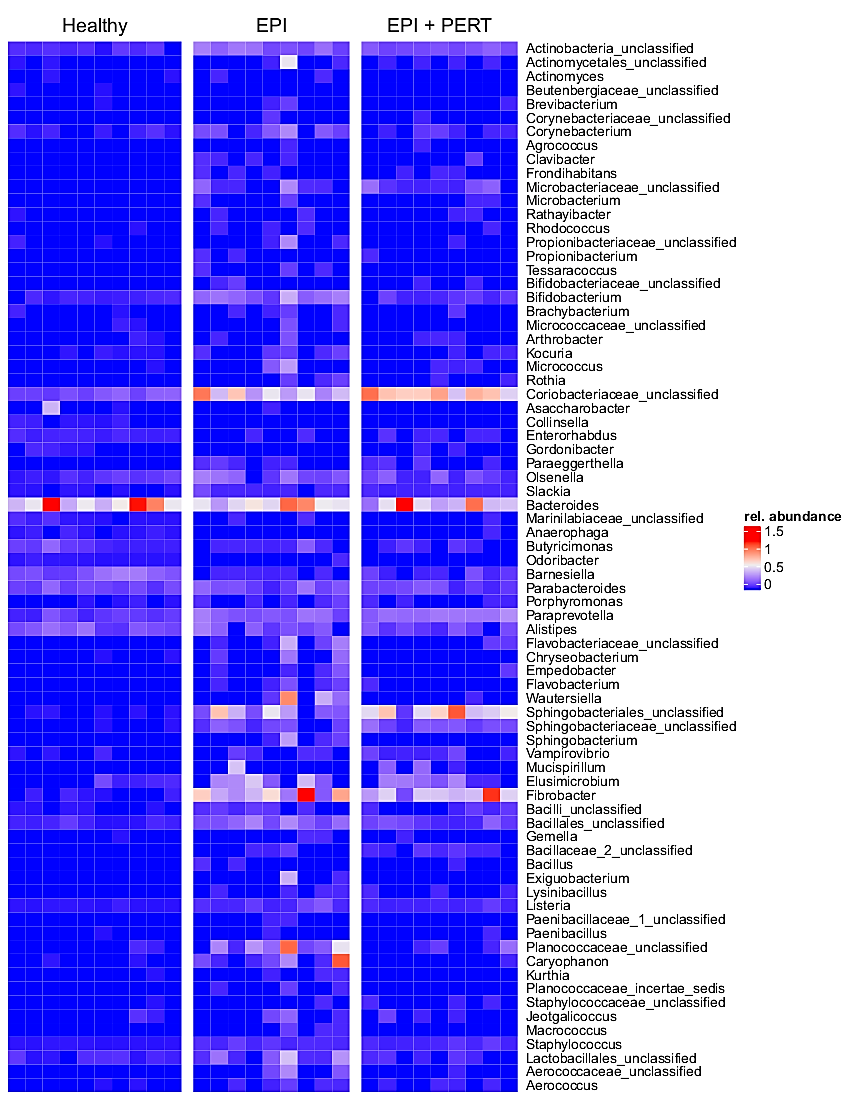


**Additional file 2A.** Heat map of the realtive abundance of microbial genera in healthy Göttingen minipigs (Healthy, n = 10), in Göttingen minipigs with induced exocrine pancreatic insufficiency without treatment (EPI, n = 9) or after at least 28 day pancreatic enzyme replacement therapy (EPI + PERT, n = 9). Red color represents highest relative abundance, blue color represents lowest realtive abundance. Asterisk marks significant differences between the abundance in EPI- and EPI + PERT- animals (α = 0,05).


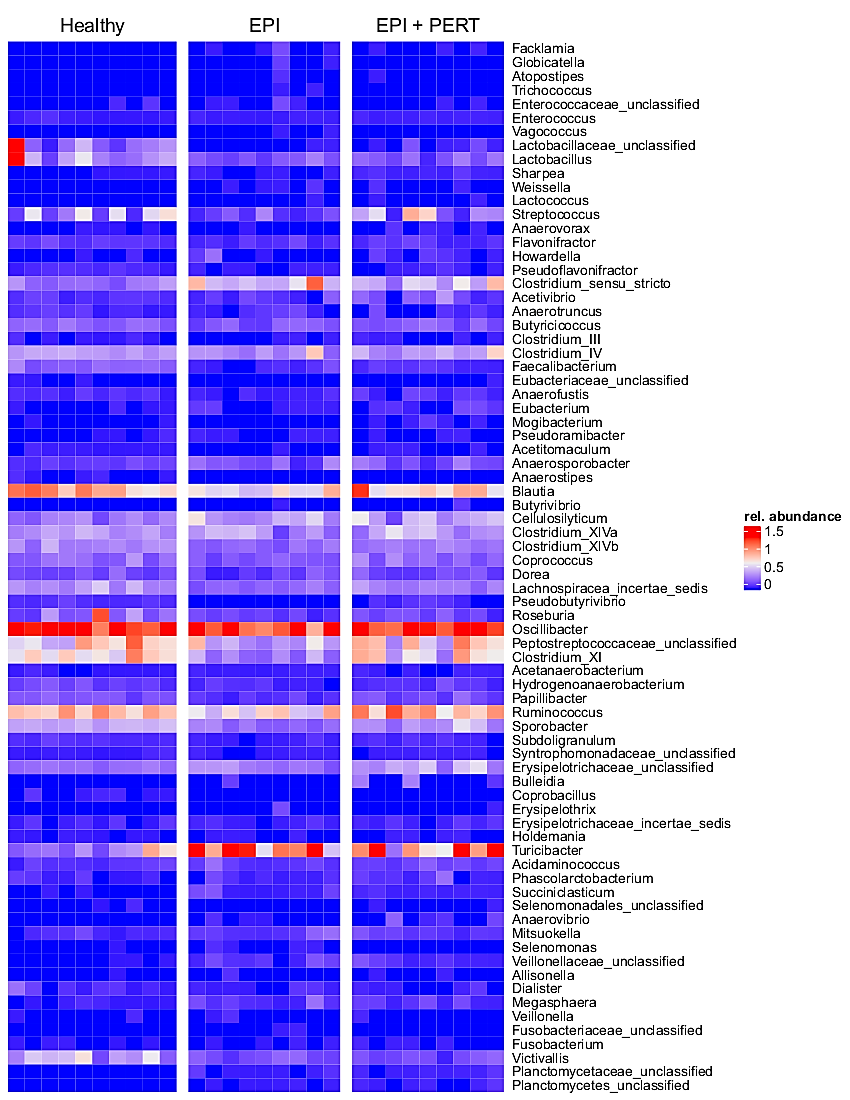


**Additional file 2B.** Continued heat map of the realtive abundance of microbial genera in healthy Göttingen minipigs (Healthy, n = 10), in Göttingen minipigs with induced exocrine pancreatic insufficiency without treatment (EPI, n = 9) or after at least 28 day pancreatic enzyme replacement therapy (EPI + PERT, n = 9). Red color represents highest relative abundance, blue color represents lowest realtive abundance. Asterisk marks significant differences between the abundance in EPI- and EPI + PERT- animals (α = 0,05).

**
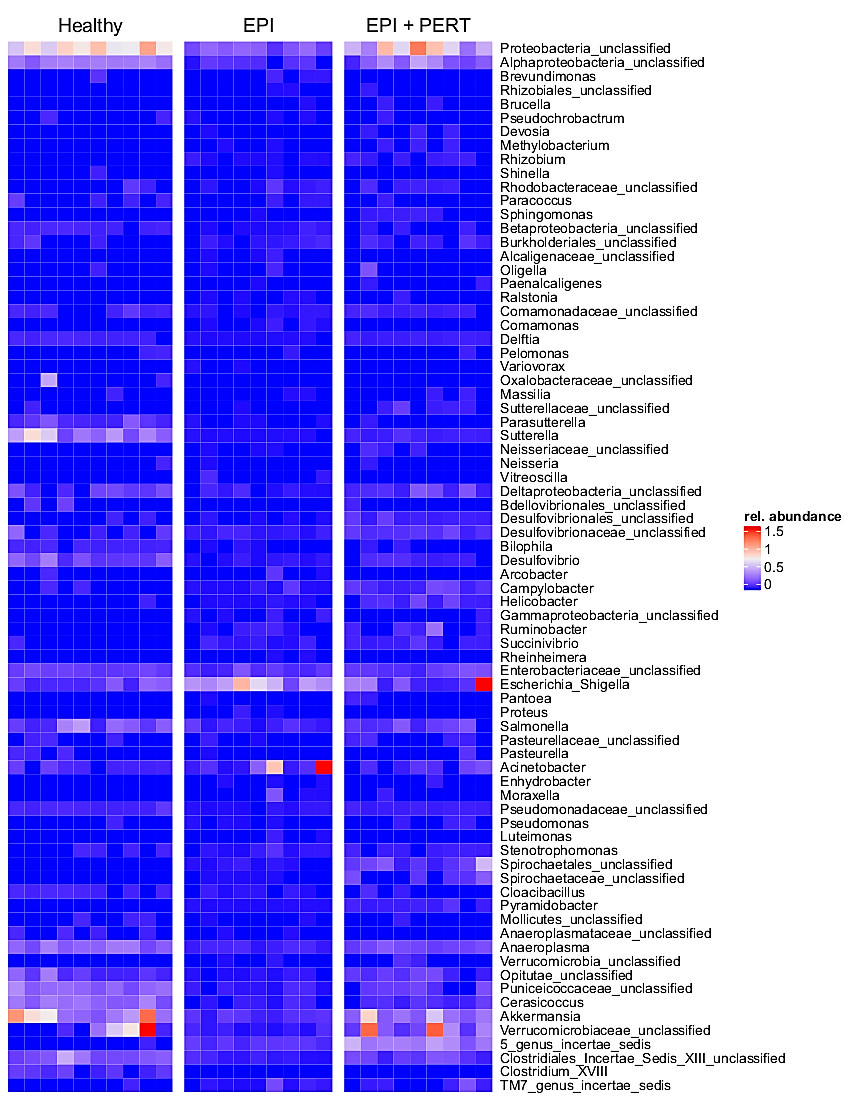
**

**Additional file 2C.** Continued heat map of the realtive abundance of microbial genera in healthy Göttingen minipigs (Healthy, n = 10), in Göttingen minipigs with induced exocrine pancreatic insufficiency without treatment (EPI, n = 9) or after at least 28 day pancreatic enzyme replacement therapy (EPI + PERT, n = 9). Red color represents highest relative abundance, blue color represents lowest realtive abundance. Asterisk marks significant differences between the abundance in EPI- and EPI + PERT- animals (α = 0,05).


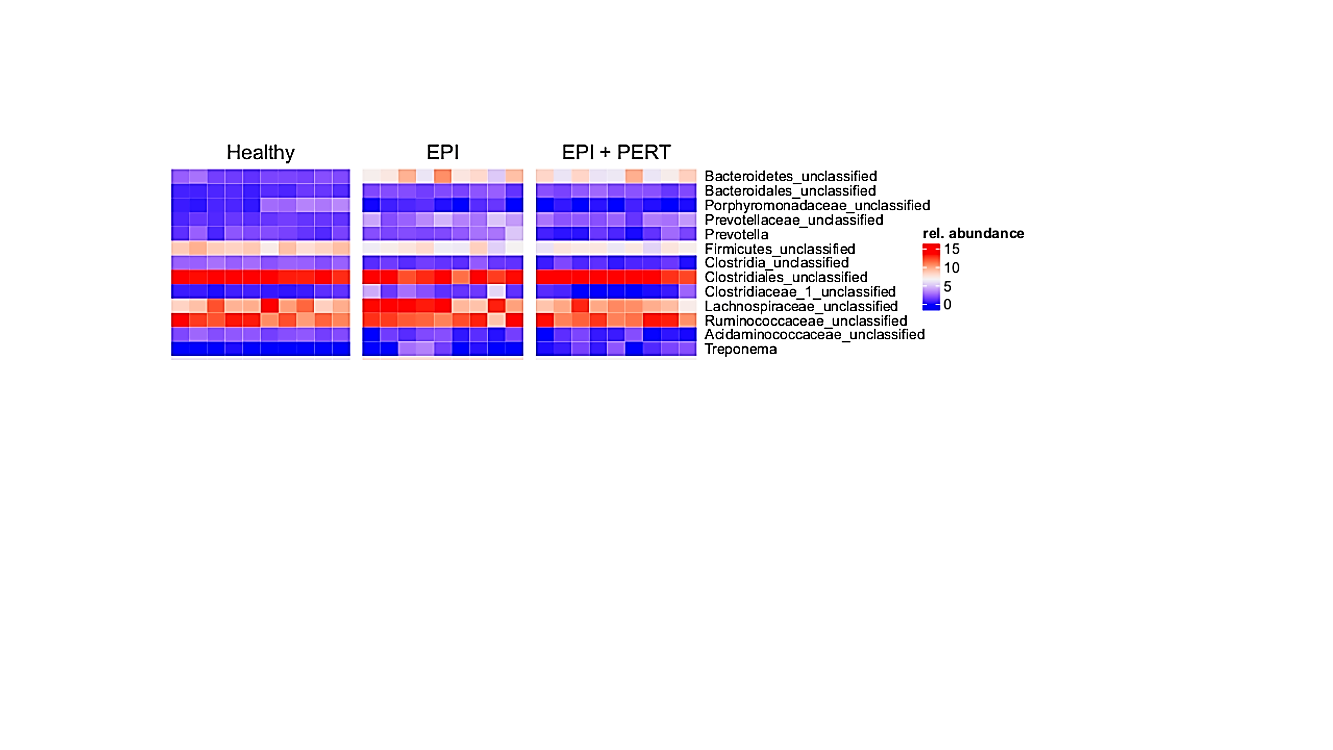


**Additional file 2D.** Continued heat map of the realtive abundance of microbial genera in healthy Göttingen minipigs (Healthy, n = 10), in Göttingen minipigs with induced exocrine pancreatic insufficiency without treatment (EPI, n = 9) or after at least 28 day pancreatic enzyme replacement therapy (EPI + PERT, n = 9). Red color represents highest relative abundance, blue color represents lowest realtive abundance. Asterisk marks significant differences between the abundance in EPI- and EPI + PERT- animals (α = 0,05).
